# Supplementary material for: Patterns of Intron Gain and Loss in Fungi
Source: PLoS Biol. 2004 Nov 30;2(12):e422. doi: 10.1371/journal.pbio.0020422 (PMC532390; doi:10.1371/journal.pbio.0020422)
Supplement: Table S1 — Also available at http://genes.mit.edu/NielsenEtAl/. (4.3 MB ZIP). [file pbio.0020422.st001.zip › NielsenEtAl/html/772.html]

AN8182.1.NCU03795.1.MG07466.1.FG07380.1


```
 CLUSTAL W (1.82) Multiple Sequence Alignments - Introns Inserted


Sequence 1: MG07466.1	385 aa
Sequence 2: FG07380.1	385 aa
Sequence 3: NCU03795.1	385 aa
Sequence 4: AN8182.1	399 aa
Alignment Length: 400 aa
Number Identitical Residues: 330 aa
Alignment Score (without introns) 13108


MG07466.1 	MAPAATESASPIGIAN~LPNQR~HKIVAKRGAAFTIM0VAGESGLGKTTFINTLFSTTIK
NCU03795.1	MAPATTESASPIGIAN~LPNQR~HKIVAKRGAAFTIM0VAGESGLGKTTFINTLFSTTIK
FG07380.1 	MASAAAENASPIGIAN~LPNQR~HKIVAKRGAGFTIM0VAGESGLGKTTFINTLFSTTIK
AN8182.1  	MAPVN-ETASPIGIAN0LPNQR2HKIVAKRGAAFTIM0VAGESGLGKTTFINTLFSTTIK
          	**..  *.******** ***** *********.**** **********************

MG07466.1 	NYADHKRRHQKQIDKTVEIEITKAELEEKFFKV1RLTVIDTPGFGDYVNNRDSWMPIIEF
NCU03795.1	NYADHKRRHQKQVDKTVEIEITKAELEEKFFKV1RLTVIDTPGFGDYVNNRDSWMPIIEF
FG07380.1 	NYADHKRRHQKQVDKTVEIEITKAELEEKFFKV1RLTVIDTPGFGDYVNNRDSWMPIIEF
AN8182.1  	NYADHKRRHQKQVDRTVEIEITKAELEEKFFKV1RLTVIDTPGFGDYVNNRDSWQPIIEF
          	************:*:****************** ******************** *****

MG07466.1 	LDDQHESYMLQEQQPRRQDKIDLRVHACLYFIRPTGHTLKPLDIEVMKRLCSRVNLIPVI
NCU03795.1	LDDQHESYMLQEQQPRRQDKIDLRVHACLYFIRPTGHTLKPLDIEVMKRLCSRVNLIPVI
FG07380.1 	LDDQHESYMLQEQQPRRQDKIDLRVHACLYFIRPTGHTLKPLDIEVMKRLCSRVNLIPVI
AN8182.1  	LDDQHESYMLQEQQPRRTDKIDMRVHACLYFIRPTGHTLKPLDIEVMKRLSSRVNLIPVI
          	***************** ****:***************************.*********

MG07466.1 	AKADTLSPADLARFKQR0IRAVIDAQSIKIYQPPVEEDDEAAAQHARSLMAAMPFAVIGS
NCU03795.1	AKADTLSPADLARFKSR0IRAVIEAQGIKIYQPPIEEDDEAAAQHARSLMAAMPFAVIGS
FG07380.1 	AKADTLSPADLAKFKQR0IVSVIEAQNIKIYQPPIEEDDEAAAQHARSLMNAMPFAVIGS
AN8182.1  	AKADTLSPADLSRFKQR0IQAVIEAQGIKIYTPPIEEDDETAAAHARSLMAAMPFAVIGS
          	***********::**.* * :**:**.**** **:*****:** ****** *********

MG07466.1 	EKDVKTGDGRIVKGRQYSWGVAEVENEDHCDFKKLRSILIRTHMLDLIHTTEELHYEAYR
NCU03795.1	EKDVKTSDGRIVKGRQYSWGVAEVENEEHCDFKKLRSILIRTHMLDLIHTTEELHYEAYR
FG07380.1 	EKDVKTGDGRIVKGRQYSWGVAEVENEDHCDFKKLRSILIRTHMLDLIHTTEELHYEAYR
AN8182.1  	EKDVKTNDGRVVKGRQYAWGVAEVEDEEHCDFKKLRSILIRTHMLDLIHTTEEQHYEAYR
          	******.***:******:*******:*:************************* ******

MG07466.1 	AQQMETRKFGEARPRKLDNPKFKEEEEALRKRFTEQVKIEEQRFRQWEQKLIAERDRLNK
NCU03795.1	AQQMETRKFGEARPRKLDNPKFKEEEEALRKRFTEQVKIEEQRFRQWEQKLIAERDRLNK
FG07380.1 	AQQMETRKFGEARPRKLDNPKFKEEEEALRKRFTEQVKIEEQRFRQWEQKLISERDRLNK
AN8182.1  	AQQMETRKFGEARPRKLDNPKFKEEEENLRKRFTDQVKLEESRFRQWEQKLIAERDRLNK
          	*************************** ******:***:**.**********:*******

MG07466.1 	DLEHTHAT--------IKQLEGELEQMQGSAARSHGRR-------
NCU03795.1	DLEQTHAQ--------IKSLEMELESLQGNAVRSHGRR-------
FG07380.1 	DLEQTHAQ--------IKQLEQELEQMQGSAVRSHGRR-------
AN8182.1  	DLEATHAAYVPEFSVYARSSIVGLTWIQHQSSRERDRKPARLIDS
          	*** ***   .. :   :.    *  :* .: *.:.*:.:   .:
```
